# Curcumin supplementation improves the clinical outcomes of patients with diabetes and atherosclerotic cardiovascular risk

**DOI:** 10.1038/s41598-025-09783-5

**Published:** 2025-08-04

**Authors:** Omar M. El-Rakabawy, Amal A. Elkholy, Amr A. Mahfouz, Mona M. Abdelsalam, Lamia M. El Wakeel

**Affiliations:** 1 Department of Clinical Pharmacy, Faculty of Pharmacy, October 6 University, 6 October City, 12585 Giza, Egypt; 2 Department of Clinical Pharmacy, Faculty of Pharmacy, Ain Shams University, Cairo, 11566 Egypt; 3 Department of Internal Medicine, Endocrinology Department, National Institute of Diabetes and Endocrinology, Cairo, Egypt; 4 Department of Internal Medicine, Endocrinology and Metabolism, Faculty of Medicine, Ain Shams University Hospital, Cairo, Egypt

**Keywords:** ASCVD, Curcumin, Diabetes mellitus, Hypertension, TNF-alpha, MDA, Cardiology, Endocrinology

## Abstract

Atherosclerotic cardiovascular diseases (ASCVD) significantly contribute to global mortality, especially in type 2 diabetes mellitus (T2DM), necessitating effective preventive strategies. Curcumin is proposed to lower blood pressure, glucose level, and improve lipid profiles as an adjunctive treatment. The study aimed to assess the safety and efficacy of Curcumin supplementation on clinical outcomes and ASCVD risk of T2DM patients. Seventy-two diabetic patients with an ASCVD risk score of ≥ 5% were randomly assigned to Curcumin group (500 mg Turmeric curcumin^®^ thrice daily + conventional therapy) or Control group (conventional therapy only). Curcumin significantly reduced SBP and DBP (*P* ≤ 0.001 and *P* = 0.020, respectively) and improved ASCVD risk classification (*P* = 0.004). LDL-C (*P* = 0.024), TNF-α (*P* = 0.044), and MDA (*P* = 0.028) levels decreased, while HDL-C increased (*P* = 0.024) versus control. No significant differences were found between groups regarding HbA1c, FBG, TC or TG (*P* > 0.05). Mild adverse effects were reported, including nausea (13.9%), headache (11.1%), yellow stool (11.1%), and diarrhea (5.6%). It is concluded that Curcumin improves ASCVD risk classification, lowers SBP, DBP, LDL-C, TNF-alpha, and MDA, increases HDL-C, and is well tolerated with minor adverse effects, without impacting on BMI, HR, HbA1c, FBG, TC, or TG.

## Introduction

Diabetes mellitus (DM) is among the most frequent ailments that people suffer from. The World Health Organization (WHO) estimates that approximately 422 million people worldwide are impacted by diabetes, with 1.5 million fatalities directly linked to diabetes each year, a number that consistently rises annually with the majority of diabetes-related mortalities, amounting to approximately 48%, transpiring in individuals below the age of 70^1^. Profound hyperglycemia, which manifests in the form of polyuria, polydipsia, unintended weight loss, and occasionally accompanied by polyphagia and blurred vision, represents the constellation of symptoms indicative of the severity of this condition^2^. Type 1, type 2, neonatal diabetes, gestational diabetes and steroid-induced diabetes are among numerous types of the disease^3^.

Regardless of the type of diabetes, it gives rise to a plethora of long-term macrovascular and microvascular complications such as retinopathy, which may ultimately culminate in the unfortunate loss of vision, nephropathy, which can lead to the debilitating condition of renal failure, peripheral neuropathy, which heightens the susceptibility to the formation of amputations, Charcot’s joints and foot ulcers as well as autonomic neuropathy, which can manifest in a range of distressing symptoms related to the cardiovascular, genitourinary, gastrointestinal and sexual dysfunction^4^.

Atherosclerotic cardiovascular disease (ASCVD), which includes coronary heart disease, stroke, and a number of peripheral artery diseases, is reigning as the leading cause of mortality worldwide^5^. Underlying mechanisms of atherosclerosis occur in various arteries of the body, including coronary arteries, cerebral, iliac, femoral artery and aorta. The complicated process begins with endothelial activation followed by a certain events including formation of lipids that is mainly cholesterol and its esters, fibrillary elements formation, as well as calcification of the affected vessels^6^. Through these pathological changes, vasoconstriction takes place and hence increasing the risk for early-onset cardiovascular disease^7^. Additionally, diabetes mellitus, hypertension, hyperlipidemia, smoking and obesity are among the risk factors for ASCVD^8^. Thus, for reduction of the ASCVD risks, there is a necessity for a comprehensive approach that includes changes in the patient’s lifestyle, as well as controlling of lipid profile, blood pressure, and glucose levels altogether^9^.

In essence, the variation in risk concerning atherosclerotic cardiovascular disease is distinctly noticeable among people who typically maintain good health and those diagnosed with clinical ASCVD^10^. The current approach to the primary prevention of ASCVD incorporates risk evaluation as a fundamental requirement. From the existing US-derived risk scores, Pooled cohort equations which assigns scores based on factors such as age, gender, blood pressure, concentration of total and HDL cholesterol, smoking status, history of diabetes and current status of antihypertensive medication use^11^. Upon summing up the allocated points, a 10-year risk estimate for cardiovascular disease (CVD) is produced. In addition, the assessment is divided into four risk categories which includes low risk (< 5%), border line risk (5-7.5%), intermediate risk (7.5–20%) and high risk categories (> 20%)^10^.

The American College of Cardiology (ACC) recommends the patients classified as high risk to employ both pharmacologic and lifestyle therapies, whereas those at low risk are often advised to modify their lifestyle solely^12^. Alongside their adverse effects, both statins and antihypertensive therapies exhibit varying patient responses. Consequently, looking for a safe and efficacious intervention for controlling these risk factors might play a crucial role in the prevention of ASCVD.

Numerous conditions such as hypertension, diabetes mellitus and lipid abnormalities have been shown to be significantly influenced by oxidative stress and inflammation as well. Those two closely related biological processes are also implicated in the pathogenesis of several diseases. It has been hypothesized that reactive oxygen species (ROS), a group of highly reactive compounds derived from oxygen metabolism, may contribute to the development of hypertension through disruption of the delicate balance of the vascular wall’s homeostasis. In various studies using experimental models and actual hypertensive patients, significant correlation has been observed between elevated ROS production, decreased nitric oxide (NO) concentrations, and reduced antioxidant bioavailability, all of which appear to be associated with the progression and prognosis of hypertension^13^.

Moreover, malondialdehyde (MDA), a by-product of peroxidation of polyunsaturated fatty acids within the cell, exhibits a high level in patients suffering from hypertension^14^. According to previous findings, oxidative stress influences beta-cell function in diabetic patients through several distinct biochemical pathways. It significantly lowers insulin synthesis, hinders proinsulin vesicle inclusion into the plasma membrane, and lowers their exocytosis in response to blood glucose levels^15^. Furthermore, it has been revealed that this occurs early in the development of hyperlipidemia, suggesting that increasing antioxidant supply to people with higher blood lipid levels may help prevent the progression of the condition^16^.

Endothelial dysfunction, denoting an impaired function at the endothelium, gives a possible mechanism to how inflammation may contribute to the initiation of hypertension. This dysfunction is recognized by decreased nitric oxide (NO) bioactive properties that play a potential role in regulating blood vessel tone and systemic blood pressure. Hypertension has been closely linked to diminishing NO bioactivity.

Conversely, inflammation can affect the rate of synthesis and breakdown of various compounds that have the capability to either constrict or dilate blood vessels, including NO^17^. The complicated interplay between inflammation and elevated blood pressure was thoroughly investigated, and studies revealed that chronic inflammatory conditions including hypertension and diabetes mellitus, have been closely associated with raised circulating tumor-necrosis-factor-alpha (TNF-α), a cytokine enrolled in diverse physiological processes^18^. This elevation of TNF-α during chronic inflammation aggravates endothelial dysfunction, thus contributing to the initiation and worsening of hypertension.

Furthermore, inflammation has been demonstrated to adversely influence cholesterol metabolism and homeostasis, which can significantly compromise cardiovascular health. Evidence from studies suggests that inflammation reduce the levels of HDL, apolipoproteins, enzymes, and antioxidants capacity that are fundamental in establishing normal lipid profile and prevent the development of ASCVD^19^. As a result, approaches focused on mitigating inflammation and oxidative stress may reduce the incidence of hypertension and its related consequences, subsequently lowering the overall risk of ASCVD.

In the contemporary era, there was a substantial drive towards the application of natural products in the desire to get the stated improvements in various medical conditions. Among this group of products, Curcumin, a polyphenolic compound, is discovered within turmeric, the rhizomatous part of the *Curcuma longa species*^20^. It is thought to be 75% of the curcuminoids extracted from turmeric. A multitude of investigations have incontrovertibly demonstrated that curcumin possess an anti-inflammatory, antioxidant, and anticancer properties, thereby conferring potential benefits to the well-being of the human race^21-23^.

There has been much scientific debate about the effects of curcumin on blood pressure. Several studies have been conducted on blood pressure lowering ability of curcumin, with somewhat conflicting results. Some studies did confirm the hypothesis that curcumin can effectively lower blood pressure, while others failed to show a significant correlation between the two variables^24^. It is widely believed that curcumin exerts its regulatory effect on blood pressure through various mechanisms. These include inhibition of the proliferation and migration of vascular smooth muscle cells, suppression of vascular contraction, enhancement of endothelial cell dysfunction, and inhibition of the renin-angiotensin system^25^. However, because of differences in dosage, sample sizes, and experimental designs, the majority of the available studies were inconclusive.

Another study^26^ showed that curcumin has a potential role in mitigating oxidative stress and inflammation. It was also postulated^27^ that curcumin may be beneficial in diabetes as it lowers glycated hemoglobin (HbA1C), improves fasting blood glucose (FBG), and may hence lessen the complications related to diabetes. Furthermore, the evidence demonstrates that curcumin can effectively modulate the lipid profile of individuals with dyslipidemia by reducing total cholesterol (TC) and low-density lipoprotein cholesterol (LDL-C), while concurrently enhancing their levels of high-density lipoprotein cholesterol (HDL-C)^28^. There is a notion that curcumin may play a beneficial role in lowering the risk of atherosclerotic cardiovascular disease.

Therefore, the aim of this study was to assess the safety and efficacy of curcumin supplementation on clinical outcomes, oxidative stress, and inflammation among diabetic patients at risk for ASCVD.

## Patients and methods

### Design & setting

The study was a prospective, open label, randomized controlled trial. Participants were recruited from the National Institute of Diabetes and Endocrinology (NIDE) and the Endocrinology Department Clinic, Demerdash Hospital, Ain Shams university hospital, Cairo, Egypt, from July 2023 to Mar 2024.

### Ethical approval

This study was conducted following the guidelines of the Declaration of Helsinki and received approval from the Ethics Committee of College of Pharmacy at Ain Shams University, located in Cairo, Egypt (RHDIRB2020110301 REC#120), as well as from the National Institute of Diabetes and Endocrinology. The study was registered on Clinicaltrials.gov (NCT ID: NCT05753436) on 03/03/2023. A written informed consent was obtained from each participant.

### Patients

Patients enrolled in the study were aged 50 to 74 years old with a calculated 10-year ASCVD risk score of at least 5%, and who fulfilled the following inclusion criteria: previously or recently diagnosed with hypertension and dyslipidemia, diagnosed with diabetes mellitus type 2 receiving treatment with insulin or oral hypoglycemic agents while maintaining a HbA1c level below 10%, and have the willingness to provide an informed consent. Criteria for exclusion were hereditary or acquired bleeding disorders, cholelithiasis, biliary tract or gall bladder ailments, other active liver diseases, pregnancy, breastfeeding, and the use of oral hypoglycemic agents that influence cardiovascular risk (Sodium-glucose co-transporter 2 (SGLT2) inhibitors, Glucagon-like peptide 1 (GLP-1) analogues).

Additionally, clinical ASCVD patients (transient ischemic attack (TIA), stroke, peripheral arterial disease, coronary or another arterial revascularization, myocardial infarction, stable or unstable angina) were excluded.

Eligible participants were randomly allocated to one of the two groups: Group I (Curcumin group, *n* = 36): received conventional therapy in addition to Turmeric curcumin 500 mg three times daily following each meal, for 14 weeks. Group II (Control group, *n* = 36): received their conventional therapy for 14 weeks.

The selected dose of 1500 mg/day was based on several prior human clinical trials that have used similar or higher doses of curcumin in capsule form with established safety profiles, including^28^. Additionally, Phase I dose-escalation study^29^ demonstrated that doses up to 8,000 mg/day of curcumin in capsule form were well tolerated in humans.

Conventional therapy included target medication for diabetes, hypertension, and dyslipidemia as per the most recent guidelines. Specifically, SGLT2 inhibitors (empagliflozin and dapagliflozin) and GLP-1 analogues (liraglutide and semaglutide) are glucose-lowering drugs that were excluded from the study to avoid overestimating curcumin’s cardiovascular benefit. SGLT2 inhibitors lower ASCVD risk by enhancing endothelial function, promoting natriuresis, and reducing blood pressure and weight. GLP-1 receptor agonists provide cardiovascular protection through anti-inflammatory mechanisms, improved glycemic control, and beneficial effects on lipid metabolism and weight.

A simple randomization process was implemented, allowing the patients to choose between even and odd numbers for the purpose of allocating groups.

Puritans Pride Turmeric curcumin^®^ 500 mg supplement which consisted of Turmeric (Curcuma longa) root 450 mg and Turmeric extract (Curcuma longa root 50 mg) standardized to ensure a 95% concentration of curcuminoids.

In order to tackle the issue of low absorption rate and limited bioavailability, participants were explicitly advised to consume the capsules alongside their main meals. They were also cautioned against changing their dietary preferences, exercise routines, or medication usage throughout their time in the trial. The compliance of patients and the surveillance of potential adverse effects were evaluated through the implementation of a visit every four weeks, in addition to weekly telephone communication throughout the duration of the study. Furthermore, to ascertain complete ingestion of the supplement, participants were instructed to hand in the emptied supplement containers after each visit.

### Methods

All patients in both groups were subject to a thorough evaluation including data collection, laboratory and clinical evaluation at baseline and at the end of the study.

*Baseline data collection* included patients’ demographics, current and past medical and medication histories. Computation of the 10 year ASCVD risk was assessed for all patients at baseline and at the end of the study utilizing the Pooled Cohort Equations (available online at tools.acc.org/ASCVD-Risk-Estimator-Plus)^30^.

To precisely evaluate the risk, essential parameters were obtained including gender, age, total cholesterol, HDL cholesterol, systolic blood pressure, diabetes history, smoking habits, and the contemporary status of antihypertensive management. The resulting scores were summed up to yield a decadal estimation representing a 10-year estimate of the risk of developing atherosclerotic cardiovascular disease. Other anthropometric measures such as weight (Kg), height (m), and body mass index (BMI) were also assessed. Social history and lifestyle factors, including tobacco use, alcohol intake, cannabis abuse, chemical ingestion, physical activity, and dietary habits (both healthy and fast foods), were evaluated.

With respect to lifestyle habits, factors such as smoking status, dietary patterns which are categorized into healthy foods, predominantly comprising fruits, vegetables, grains, and lean proteins, and unhealthy foods, which primarily consist of fried items, processed snacks, and high-fat meals. Moreover, exercise was defined as engaging in a minimum of 30 min of walking per day.

*Laboratory evaluations* were performed for both groups at baseline and after 14 weeks. Blood samples were withdrawn from all patients for assessment of the various parameters. Total cholesterol (TC), HDL cholesterol, and triglyceride levels (TG) were performed utilizing enzymatic colorimetric techniques. These methods employed high-quality reagents obtained from Spinreact (Girona, Spain). LDL cholesterol was computed using the Friedewald equation (TC (mg/dL) − HDL-c (mg/dL) − TG (mg/dL)/5)^31^. Glycated hemoglobin and fasting blood glucose were also determined using the commercial kit Spinreact (Girona, Spain). To quantify the serum TNF-α, an enzyme-linked immunosorbent assay (ELISA) kit provided by ELK Biotechnology Co., Ltd (Wuhan, China) was utilized. On the other hand, the determination of MDA levels was carried out through the utilization of a colorimetric technique using a kit manufactured by Biodiagnostic (Giza, Egypt).

*Clinical evaluations*. At each clinic visit, patients were allowed to sit relaxed for 30 min, then blood pressure was measured in both arms. From the higher arm, three readings were taken 5 min apart and the last 2 were averaged. The pulse was also taken and reported.

*Adverse effect reporting*. To track any side effects, an educational leaflet was given to each patient with a list of possible adverse effects (nausea, diarrhea, headaches, skin rashes, and yellow feces) for self-reporting at occurrence.

### Sample size calculation

The sample size was calculated using G* Power v.3.1.9.2 (Institut für Experimentelle Psychologie, Heinrich Heine Universität, Düsseldorf, Germany). According to a previous study^32^ individuals who were administered a placebo experienced a mean change of -1.6 ± 7.7 (mg/dl) in their HDL levels, while those who received curcumin demonstrated a mean change of 4.5 ± 8.9 (mg/dl). These observations necessitate a minimum sample size of 31 subjects in each group, with an alpha level of 0.05 and a power of 80%. In order to account for any potential loss to follow-up, the sample size was increased by 15% to encompass 36 subjects in each group, resulting in a total sample size of 72 subjects.

### Statistical methods

Statistical analysis was done using IBM SPSS^®^ Statistics version 26 (IBM^®^ Corp., Armonk, NY, USA). Numerical data was expressed as mean and standard deviation or median and range as appropriate. Qualitative data was expressed as frequency and percentage. Pearson’s Chi-square test or Fisher’s exact test was used to examine the relation between qualitative variables. Comparison of quantitative variables between two groups was done using either Student t-test for parametric data or Mann-Whitney test for non-parametric numerical data. Comparison between two consecutive measures of numerical variables were done using either paired t-test or Wilcoxon Signed Ranks test (non-parametric paired t-test). While comparison between more than two consecutive measures of numerical variables were done using analysis of variance (ANOVA) with repeated measures. Correlation between numerical variables was tested using Spearman-rho correlation. Due to multiple comparisons, p-value was corrected using Bonferroni method. All tests were two-tailed and P-value < 0.05 was considered significant.

## Results

**Fig. 1:**
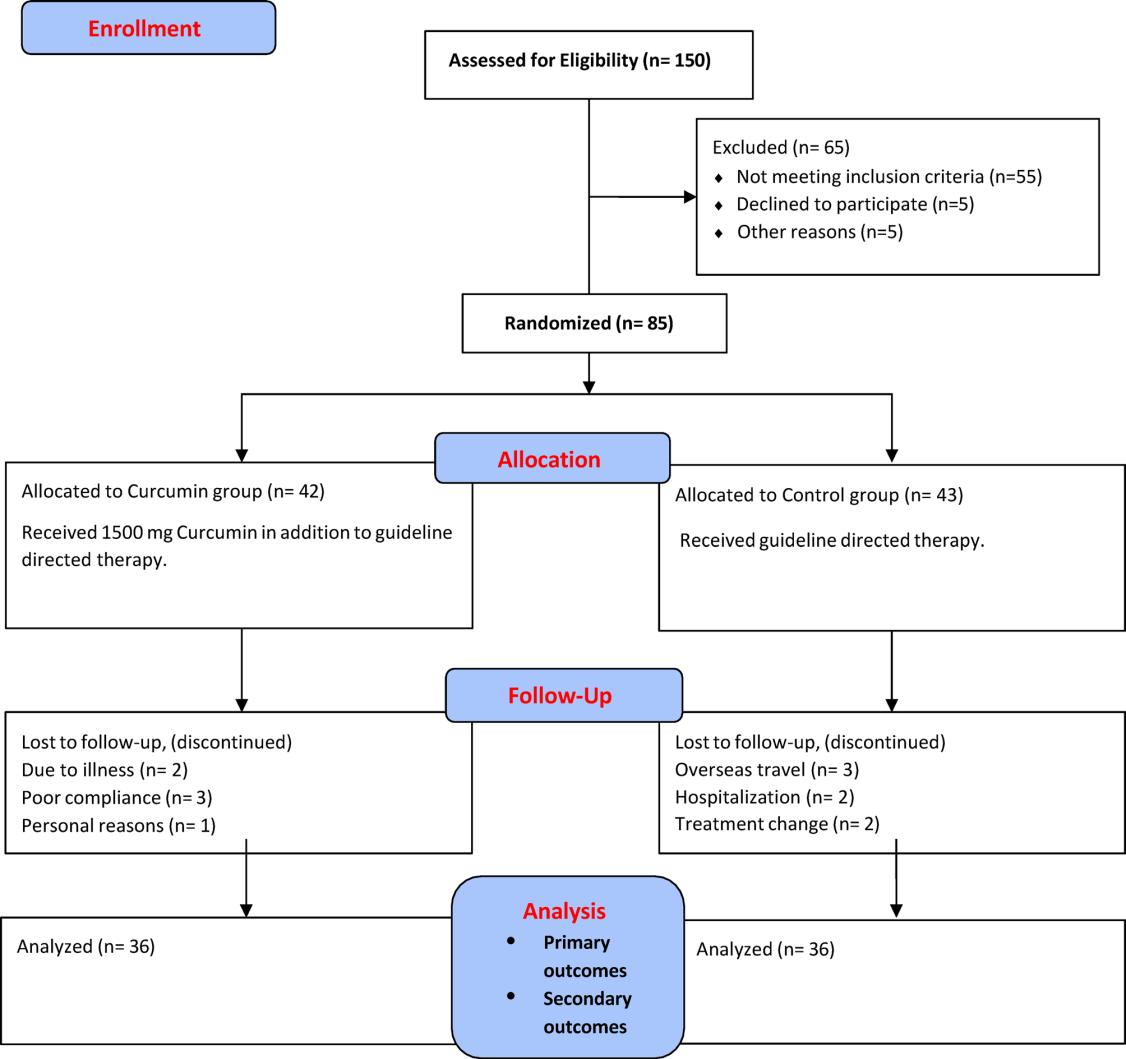
CONSORT Flow Diagram.

Out of a total of 150 patients screened, only 85 patients fulfilled the inclusion criteria and were randomly allocated into either the Curcumin group (42), or the Control group (43). Six patients in the curcumin group and seven patients in the control group were compelled to withdraw from the study. Finally, a total of 72 patients, 36 in each group, continued the study. The CONSORT flow diagram is illustrated in **Fig. **1.

### Demographics, histories and clinical characteristics

At baseline, both groups were comparable in terms of demographic characteristics, body composition metrics (body weight and BMI), lifestyle habits (smoking, diet, exercise, consumption of alcohol or recreational drugs), medical histories, duration of diseases, or drugs utilized. None of the participants in either groups reported the consumption of alcohol or the use of recreational drugs (refer to Table 1). In terms of body composition metrics, comparative analyses between the two groups revealed no statistically significant differences both at the initiation and at the end of the trial, and also within each group.

**Table 1:** **Comparison of demographic data and clinical characteristics between the two groups.** Yrs: years; BMI: body mass index; HTN: hypertension; DM: diabetes mellitus; DLD: dyslipidemia disease; ACE: angiotensin-converting enzyme; ARB: angiotensin receptor blockers; CCB: calcium channel blocker; S.D: standard deviation. (#): unpaired t test; (##): paired t test; ($): Chi-Square test; (@): Mann Whitney U test. P value 1 is for comparison variables between the two groups; P value 2 is for comparison variables in one group; *P value < 0.05 is considered significant.

Parameter	Control group: *N* (36)	Curcumin group: *N* (36)	*P* value 1
**Age (yrs): Mean ± S.D**	60.9 ± 6.6	59.8 ± 5.3	0.433#
**Height (cm): Mean ± S.D**	155.8 ± 7.6	158.9 ± 6.5	0.073#
**Weight (Kg): Mean ± S.D**			
Baseline	87.3 ± 14.3	88.4 ± 13.4	0.741#
End of study	87.4 ± 15.4	87.9 ± 13.3	0.890#
**P value 2**	0.800##	0.155##	
**BMI (kg/m2): Mean ± S.D**			
Baseline	36.1 ± 6.0	35.1 ± 5.2	0.462#
End of study	36.1.3 ± 6.5	34.9 ± 5.2	0.389#
**P value 2**	0.798##	0.223##	
**Gender: N (%)**			
Male	3(8.3%)	3(8.3%)	1.00$
Female	33(91.7)	33(91.7)	
**Smokers: N (%)**	3 (8.3%)	6(16.7%)	0.478$
**Exercise: N (%)**	13(36.1%)	9 (25.0%)	0.306$
**Diet: N (%)**			
Healthy	9(25.0%)	17(47.2%)	0.050$
Unhealthy	27(75.0%)	19(52.8%)	
**Medical history: N (%)**			
**None**	35(97.2%)	31(86.1%	
**With diseases**	1(2.8%)	5(13.9%)	
*Thyroid disease*	1(2.8%)	0(0.0%)	0.199$
*Surgical operations*	0(0.0%)	2(5.6%)	
*Other diseases*	0(0.0%)	3(8.3%)	
**Duration of HTN (yrs): Median (Range)**	10 (1–29)	10 (1–40)	0.475@
**Duration of DM (yrs): Median (Range)**	15(2–35)	10(3–30)	0.163@
**Duration of DLD (yrs): Median (Range)**	4.5(1–15)	2(1–10)	0.092@
**Medications: N (%)**			
**Antihypertensives**			
*Beta blockers*	16 (44.4%)	11(30.6%)	0.224$
*Diuretics*	2(5.6%)	2(5.6%)	1.000$
*ACE inhibitors*	19(52.8%)	16(44.4%)	0.479$
*ARB&CCB*	7(19.4%)	13(36.1%)	0.114$
**Antidiabetics**			
*Insulin*	22(61.1%)	17(47.2%)	0.237$
*Insulin & Metformin*	19(52.8%)	12(33.3%)	0.096$
*Dual oral therapy*	12(33.3%)	17(47.2%)	0.230$
*Insulin & Dual oral therapy*	1(1.4.%)	2(5.6%)	0.555$
**Antihyperlipidemics**			
*Statins*	33(91.7%)	32(88.9%)	1.000$
*Ezetimibe & simvastatin*	3(8.3%)	4(11.1%)	1.000$

**Fig. 2:**
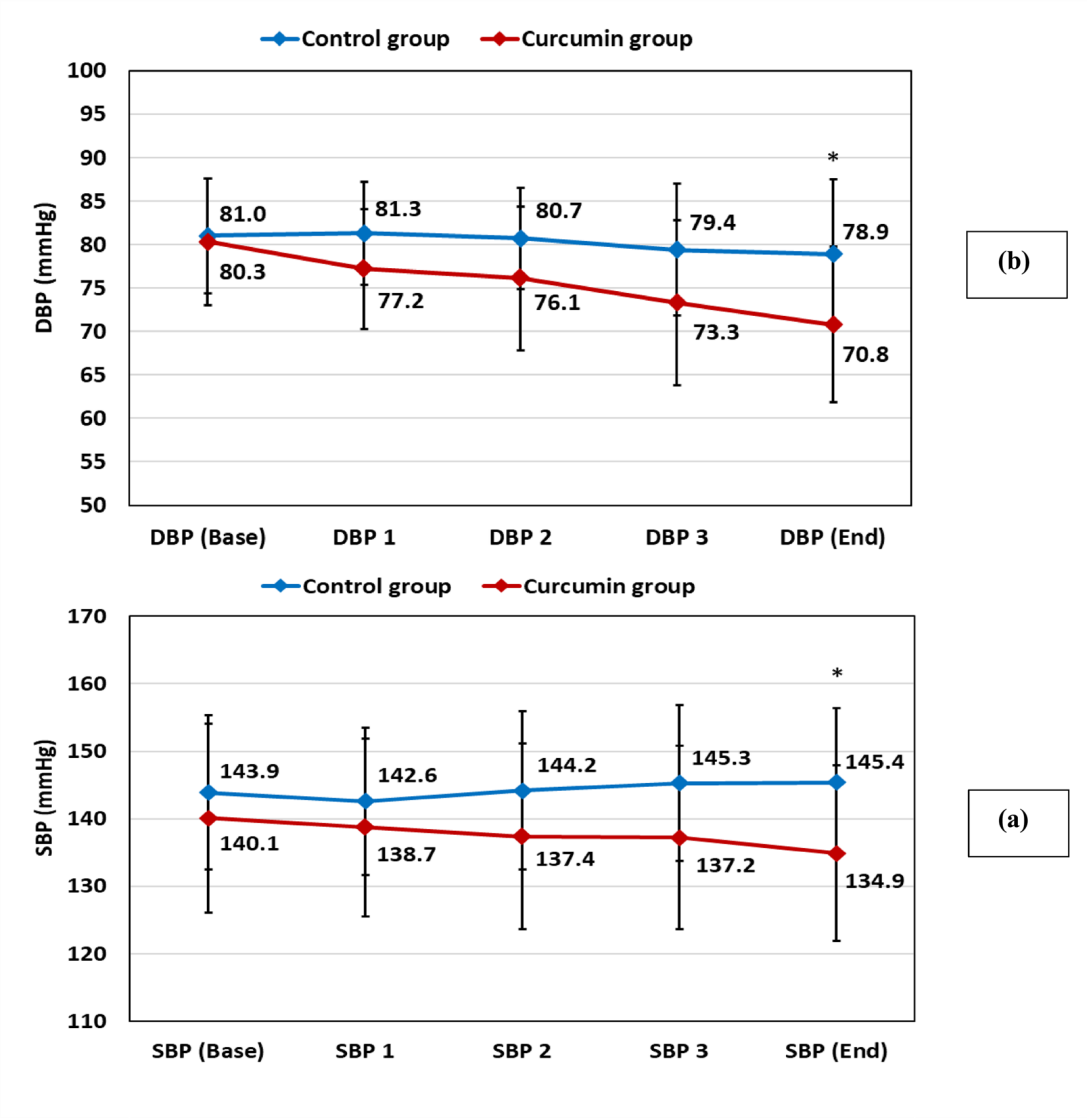
Change in Blood pressure between Curcumin and Control groups at baseline and over time. (**a**) Systolic blood pressure (SBP); (**b**) Diastolic blood pressure (DBP).

### Clinical evaluation

As shown in Table 2, at baseline, the curcumin and control groups were comparable with regards to all the clinical parameters assessed (SBP, DBP, Pulse, ASCVD score and class). At the end of the study, the mean SBP was significantly reduced in the curcumin group vs. controls (134.9 vs. 145.4, *P* < 0.001) respectively. Likewise, a discernible decline in DBP was noted in the curcumin group versus controls (70.8 vs. 78.9, *P* < 0.001) respectively, (**Fig. **2).

Moreover, within the 14 weeks’ supplementation period with curcumin the intervention group showed a statistically significant decrease in both SBP (*P* = 0.02) and DBP (*P* < 0.001) compared to their baseline values (Table 2).

**Fig. 3:**
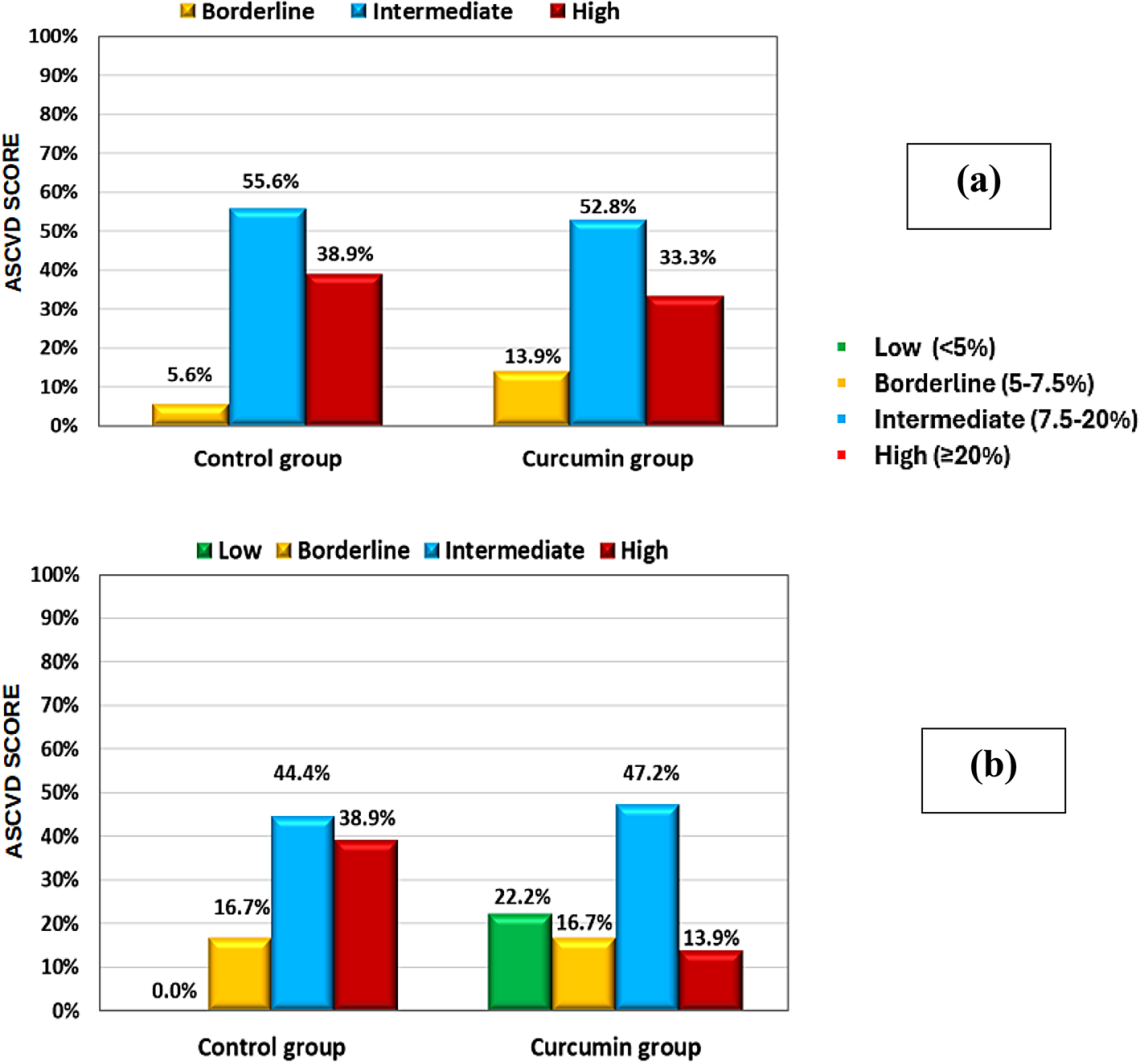
Change in ASCVD risk classes between Curcumin and Control groups at baseline (**a**) and at the end of the study (**b**).

Upon assessing the (ASCVD) risk class distribution between the two groups, a significant difference was noted at the end of the trial (*P* = 0.004). A noteworthy reduction in ASCVD risk class for the curcumin group was observed, as evidenced by a substantial shift between classes; at baseline, the high-risk category constituted 33.3%, which diminished to 13.9% by the end of the trial, while concurrently, the low-risk category, which was non-existent at the beginning, emerged at the trial’s end, representing 22.2% in the curcumin group. In contrast, alterations and shifts in risk classes were hardly observed within the control group. Moreover, there was a significant decline (*P* < 0.001) in the overall ASCVD score from baseline till study ended only in the curcumin group as detailed in Table 2; (Fig. 3).

**Table 2:** **Comparison of clinical outcomes between the two groups.** SBP: systolic blood pressure; DBP: diastolic blood pressure; P: pulse; ASCVD: atherosclerotic cardiovascular disease; (#): unpaired t test; (^): ANOVA with repeated measures; (@): Mann Whitney U test; (”): Wilcoxon signed ranks test; ($): Chi-Square test. P value 1 is for comparison variables between the two groups; P value 2 is for comparison variables in one group; *P value < 0.05 is considered significant.

Parameter	Control group: *N* (36)	Curcumin group: *N* (36)	*P* Value 1
SBP (mmHg): Mean ± S.D			
Baseline	143.9 ± 11.4	140.1 ± 14.0	0.216#
End of study	145.4 ± 11.0	134.9 ± 13.0	< 0.001*#
**P value 2**	0.195^	0.020*^	
**DBP (mmHg): Mean ± S.D**			
Baseline	81.0 ± 6.6	80.3 ± 7.3	0.673#
End of study	78.9 ± 8.6	70.8 ± 9.0	< 0.001*#
**P value 2**	0.300^	< 0.001*^	
**Pulse (bpm): Mean ± S.D**			
Baseline	81.9 ± 11.0	81.0 ± 9.3	0.704#
End of study	79.3 ± 9.8	77.6 ± 7.0	0.417#
**P value 2**	0.528^	0.420^	
**ASCVD Score (%): Median (Range)**			
Baseline	14.7(5.7–45.5)	12.8(5.6–53.7)	0.481@
End of study	12.5(5.2–45.5)	9.0(2.8–44.0)	0.104@
**P value 2**	0.285”	< 0.001*”	
**Baseline ASCVD Class: N (%)**			
*Borderline*	2(5.6%)	5(13.9%)	0.610$
*Intermediate*	20(55.6%)	19(52.8%)	
*High*	14(38.9%)	12(33.3%)	
**End of study ASCVD Class: N (%)**			
*Low*	0(0.0%)	8(22.2%)	0.004*$
*Borderline*	6(16.7%)	6(16.7%)	
*Intermediate*	16(44.4%)	17(47.2%)	
*High*	14(38.9%)	5(13.9%)	

### Laboratory parameters

**Fig. 4:**
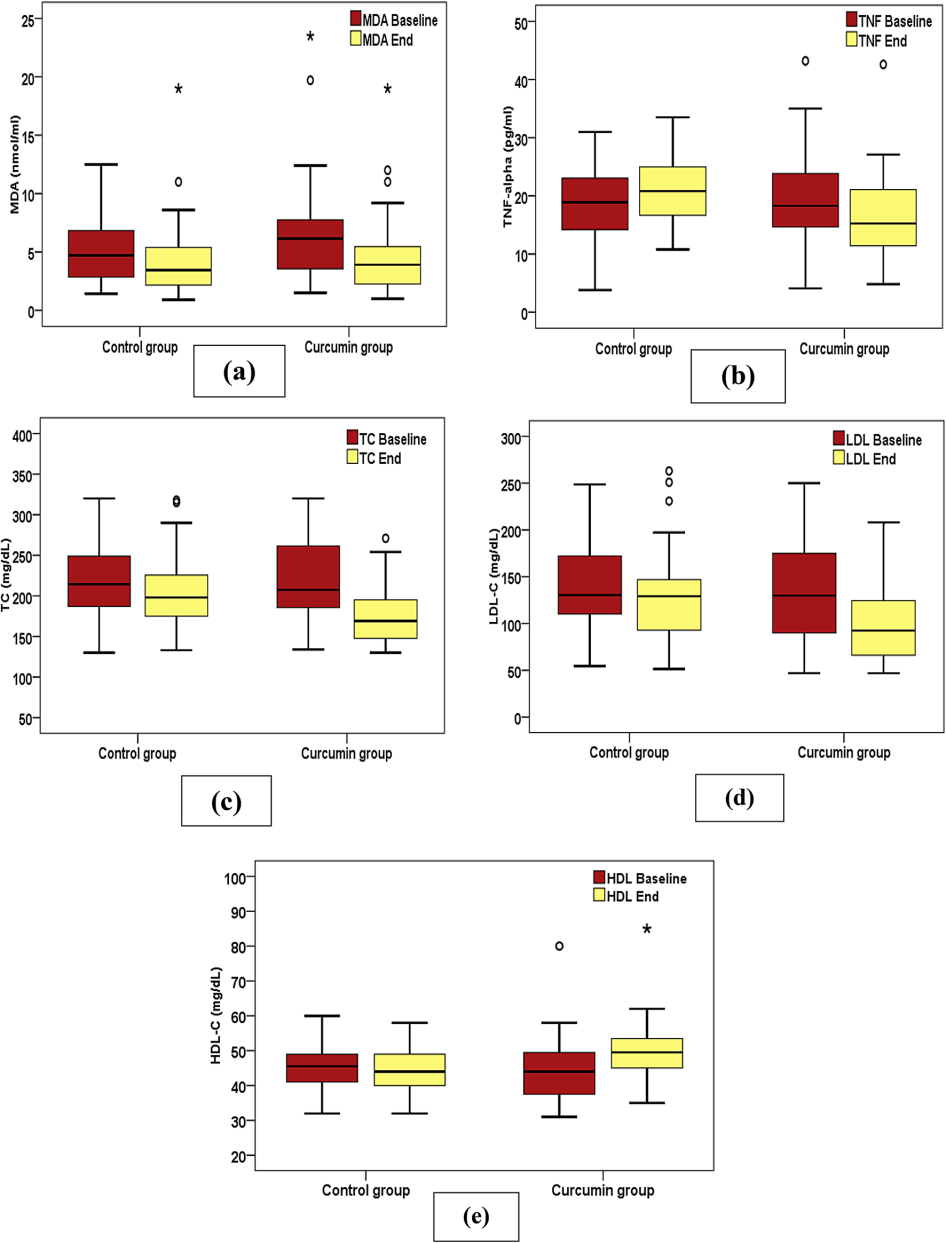
Change in Laboratory Parameters between Curcumin and Control groups at baseline and over time. (**a**) Malondialdehyde; (**b**) TNF-alpha; (**c**) Total cholesterol; (**d**) LDL-cholesterol; (**e**) HDL-cholesterol.

All laboratory parameters (HbA1c, FBG, TC, HDL-C, TG and INR) were comparable between groups at baseline. At the end of the study, the TC levels significantly declined over time within each group, with the decline being more pronounced in the Curcumin group (*P* < 0.001) versus the Control group (*P* = 0.028) (**Fig. 4c)**. Additionally, there was a significant reduction overtime in the LDL levels (*P* ≤ 0.001) and a significant elevation in HDL levels (*P* ≤ 0.001) only within the Curcumin group from baseline to the end of the study (Fig. 4d and 4e). Furthermore, at the end of the study, the Curcumin group reported lower LDL levels (*P* = 0.024) and higher HDL levels (*P* = 0.024) versus the control group.

In relation to the inflammatory (TNF-α) and oxidative stress (MDA) biomarkers, that were comparable between the groups at baseline, a significant change was observed between groups at the end of the study. TNF-α levels significantly declined overtime (*P* < 0.001) only in the curcumin group and were significantly lower (*P* = 0.044) versus controls at the end of the study (Fig. 4b). Additionally, overtime, MDA levels significantly declined (*P* = 0.028) only in the curcumin group as illustrated in Table 3; (Fig. 4a).

**Table 3:** **Comparison of laboratory parameters between groups.** HbA1c: glycated hemoglobin; FBG: fasting blood glucose; TC: total cholesterol; TG: triglycerides; LDL-C: Low-density lipoprotein cholesterol; HDL-C: High-density lipoprotein cholesterol; INR: international normalized ratio; TNF-α: tumor necrosis factor alpha, MDA: malondialdehyde; S.D: standard deviation. (#): unpaired t test; (##): paired t test; (@) Mann Whitney U test; (”): Wilcoxon signed ranks test. P value 1 is for comparison variables between the two groups; P value 2 is for comparison variables in one group; *P value < 0.05 is considered significant.

Parameter	Control group: *N* (36)	Curcumin group: *N* (36)	*P* value 1
HbA1c (mg/dl): Mean ± S.D			
Baseline	7.4 ± 1.6	7.2 ± 1.6	0.506#
End of study	7.9 ± 2.0	7.5 ± 2.0	0.482#
**P value 2**	0.140 ##	0.175##	
**FBG (mg/dl): Mean ± S.D**			
Baseline	145.9 ± 38.0	138.2 ± 41.7	0.414#
End of study	157.3 ± 44.7	154.1 ± 47.1	0.775#
**P value 2**	0.157##	0.152##	
**TC (mg/dl): Mean ± S.D**			
Baseline	218.6 ± 50.9	218.4 ± 53.2	0.991#
End of study	201.5 ± 47.7	176.1 ± 37.0	0.056#
**P value 2**	0.028*##	< 0.001*##	
**TG (mg/dl): Median (Range)**			
Baseline	137 (78–312)	150 (65–475)	0.726@
End of study	113.5 (61–349)	125 (43–341)	0.882@
**P value 2**	0.257”	0.065”	
**LDL-C (mg/dl): Mean ± S.D**			
Baseline	141.0 ± 53.5	138.1 ± 55.5	0.821#
End of study	128.5 ± 50.8	98.2 ± 39.4	0.024*#
**P value 2**	0.204##	< 0.001*##	
**HDL-C (mg/dl): Mean ± S.D**			
Baseline	45.1 ± 7.5	44.5 ± 9.1	0.767#
End of study	44.3 ± 6.6	49.6 ± 8.9	0.024*#
**P value 2**	0.389##	< 0.001*##	
**INR: Median (Range)**			
Baseline	1 (1-1.1)	1 (1.0-1.1)	0.822@
End of study	1 (1–1.0)	1 (1.0-2.1)	0.131@
**P value 2**	0.317”	0.264”	
**TNF-α: Mean ± S.D**			
Baseline	18.1 ± 6.8	20.0 ± 8.3	0.283#
End of study	20.6 ± 5.7	16.5 ± 7.4	0.044*#
**P value 2**	0.092##	< 0.001*##	
**MDA: Median (Range)**			
Baseline	4.7 (1.4–12.5)	6.1 (1.5–23.5)	0.127@
End of study	3.5 (0.9–19.0)	3.9 (1.0–19.0)	0.693@
**P value 2**	0.320”	0.028*”	

**Table 4:** **Occurrence of adverse events between groups.** ($): Chi square test. P value 1 is for comparison variables between the two groups; *P value < 0.05 is considered significant.

Adverse effects	Control group: *N* (36)	Curcumin group: *N* (36)	*P* value 1
**Nausea: N (%)**	0 (0.0%)	5 (13.9%)	0.054$
**Diarrhea: N (%)**	1 (2.8%)	2 (5.6%)	1.00$
**Headache: N (%)**	1 (2.8%)	4 (11.1%)	0.357$
**Skin rash: N (%)**	1 (2.8%)	0 (0.0%)	-
**Yellow Stool: N (%)**	0 (0.0%)	4 (11.1%)	0.115$

### Adverse events

Regarding the incidence of adverse events throughout the study, the reported side effects within the curcumin group included nausea (13.9%), headache (11.1%), yellow stool (11.1%), and diarrhea (5.6%), which represent relatively low percentages that were not statistically different from those observed in the control group (Table 4). None of the reported adverse effects required the cessation of medication or the implementation of any therapeutic interventions.

## Discussion

In contemporary times, the prevention of ASCVD has emerged as an imperative task, attributable to its classification as a significant global cause of mortality, which exhibits a progressive increase annually. With regard to the exploration of utilizing naturally derived and safe products which may prove beneficial in the management of various diseases, our study presents itself as the first clinical trial designed to evaluate the efficacy of curcumin in reducing the susceptibility to ASCVD. This investigation enabled the exploration of its influence on specific parameters encompassed in the risk assessment, namely blood pressure and lipid profile. Additionally, ancillary variables were also assessed, such as heart rate, blood glucose levels, oxidative stress and inflammatory markers.

The assessment of clinical indicators revealed that over a period of 14 weeks of curcumin supplementation at a dose 1500 mg/day, there was an overall reduction in systolic and diastolic blood pressure, LDL cholesterol, TNF alpha, MDA levels, ASCVD risk class, together with a rise in HDL cholesterol. While no significant alterations were observed in BMI, heart rate, HbA1c level, FBG level, total cholesterol and triglyceride levels.

At the outset, it is important to address a potential concern regarding baseline dietary classification. A borderline statistical difference (P = 0.05) was observed between groups in terms of general dietary habits; however, this difference was based solely on a one-time, self-reported categorization of participants' diets as “healthy” or “unhealthy” at baseline. No quantitative dietary tracking or repeated dietary assessments were conducted throughout the 14-week intervention period. Given the subjective and limited nature of this baseline assessment, and the absence of ongoing dietary monitoring, it is unlikely that this marginal difference had a substantial impact on the clinical outcomes. Additionally, other critical baseline variables influencing ASCVD risk—such as blood pressure and lipid profile —were comparable between groups, thereby supporting the overall internal validity of the findings.

In the current study, the majority of patients were obese, and the anthropometric statistical analysis revealed no significant statistical difference in weight or BMI after the administration of curcumin. These results diverge from a separate study in Italy which suggested that sustained consumption of curcumin could potentially precipitate a reduction in overall body mass over an extended timeframe^33^. This may be due to the capacity of curcumin to mitigate cortisol levels owing to its impact on the enzyme 11β-Hydroxysteroid dehydrogenase type 1. Consequently, it is anticipated that the decrease in cortisol levels could lead to an appetite suppression and ultimately weight loss.

Conversely, results from an alternative study indicated that curcumin might even cause an increase in body weight^34^. Their contrasting viewpoint hinged on the hypothesis that curcumin works via blocking TNF alpha, a cytokine known for exacerbating symptoms of cachexia including weight loss. The suggested mechanism reveals that by inhibiting TNF alpha, curcumin could actually prompt an overall increase in body weight.

Consistent with our observation, another study^35^ found no meaningful change between curcumin and placebo group, as the authors specifically focused on cancer patients in an effort to mitigate the effects of cancer anorexia–cachexia syndrome through the administration of curcumin, with the intention of increasing the overall body mass of these individuals. The authors attributed these findings to the fact that patients assigned to the curcumin group showed a 50% less demand for tube feeding interventions in comparison to those in the placebo group, thereby suggesting that patients in the placebo group might have received a sufficient amount of total caloric intake and nutritional support that surpassed their equivalents more than patients with curcumin. The discrepancy between studies could be attributed to various factors related to the disease setting with its concurrent inflammatory and oxidative stress markers’ levels as well the duration of curcumin administration and used regimens that add to the variability in pharmacokinetics and hence pharmacodynamic outcomes.

Numerous theories have been proposed concerning the association between blood pressure and curcumin, a bioactive compound with possible positive benefits on controlling systolic (SBP) and diastolic blood pressure (DBP). Empirical investigation carried out on rodent models have demonstrated that the supplementation of curcumin lowers blood pressure^36^. This physiological response is thought to be mediated through the curcumin modulatory actions on the activation of angiotensin-converting enzyme (ACE) within the central nervous system. While other researches have indicated a protective influence against increasing SBP and DBP^37,38^. The potential underlying mechanisms contributing to these observed effects in mice might be attributed to a potential augmentation in Nitric Oxide (NO) availability by up-regulating endothelial Nitric Oxide Synthase (eNOS) protein.

Transitioning to human trials, multiple studies propose that the administration of curcumin does not result in any significant difference in either SBP or DBP^39,40^. However, a systematic review has put forward the idea that supplementing with curcumin yields a positive impact on BP when taken for a period of ≥ 12 weeks, leading to a reduction in SBP but not DBP^24^. It posits that curcumin demonstrates its regulatory impact on blood pressure by employing a wide array of mechanisms. These mechanisms encompass the inhibition of vascular smooth muscle cells proliferation and migration, repressing the vascular contraction, ameliorating the dysfunction in endothelial cells, and blocking the renin-angiotensin system (RAS).

It is worth noting that this review comprises studies where none of the Randomized Controlled Trials (RCTs) were carried out on hypertensive individuals, with blood pressure being just one of the measured outcomes. Continuing this premise, our research demonstrates that the utilization of dosage 1500 mg per day of curcumin for 14 weeks exhibits a statistically significant effect on both systolic and diastolic blood pressure. This assertion may hold true as it represents the preliminary investigation into the impacts of curcumin at this specific dose and duration on actual hypertensive patients. Within our study, where we monitored the blood pressure every 4 weeks throughout the entire treatment period, no statistically significant difference in SPB and DBP was observed until the 14th week. Nevertheless, following the 10th week, the systolic blood pressure approached a point where it could be deemed a significant difference between the two studied groups (*p* < 0.06).

In the realm of cardiovascular physiology, studies conducted on the cardiac rhythm of mice were encompassed within a comprehensive systematic analysis which revealed that curcumin had no discernible effects on heart rate^41^. This particular finding aligns with the outcomes of our research, as well as with the conclusions drawn from a separate human study indicating a lack of statistically significant variance subsequent to the administration of curcumin^42^.

Beyond that, curcumin has been proven in various academic studies that it has a potential impact on different glycemic parameters, particularly HBA1c and FBG. The perception of curcumin’s efficacy depends on its direct activation of peroxisome proliferator-activated receptor-gamma (PPAR-γ) in β-cells, inhibits the production of hepatic glucose by the activation of AMP-activated protein kinase (AMPK) while also suppressing enzymes including phosphoenolpyruvate carboxykinase (PEPCK) and glucose-6-phosphatase (G6Pase). Additionally, curcumin promotes the translocation of Glucose transporter type 4 (GLUT4) which improves cellular absorption of glucose. Further, blocking of nuclear factor kappa-light-chain-enhancer of activated B cells (NF-KB) reduces insulin resistance and blood glucose levels^43,44^.

Nevertheless, these findings are not devoid of contradictions as they are set against other studies that indicate curcumin did not produce any significant effect on blood glucose level^45,46^. This discrepancy in outcomes coincides with the conclusions drawn from our study, possibly attributed to variations in dosage, study duration and the severity of the subjects’ glycemic conditions.

Hyperlipidemia, in fact, plays a key role in the pathogenesis of numerous conditions such as obesity, diabetes, inflammation, and atherosclerotic cardiovascular diseases. Several investigations have put forth optimistic assertions regarding the impact of curcumin on lipid profile, showcasing a noteworthy decrease in the concentrations of TG, TC, LDL-C, and an increase in HDL-C^47,48^.

This is contingent upon its capacity to suppress key elements involved in lipogenesis such as sterol regulatory element-binding proteins (SREBPs), 3-hydroxy-3-methylglutaryl-CoA (HMGCoA) reductase and fatty acid synthase, while also promoting the excretion of lipids and its mobilization from adipose tissue. Our research came in agreement with these aforementioned results, with the exception of total cholesterol (TC) and triglycerides (TG) where we have concluded that there is no significant difference between the two groups in their levels. Nonetheless, it is imperative to emphasize that TC exhibited a decrease within each group relative to the baseline measurements. This reduction was more pronounced in the curcumin group as opposed to the Control group. The more substantial reduction in TC within the curcumin group indicates a potentially beneficial impact of curcumin supplementation on TC levels when utilized alongside antihyperlipidemic pharmacotherapy. However, these positive results are outweighed by a clinical trial that has documented the lack of any noticeable effects of curcumin on the lipid components across diverse populations^49^. Furthermore, the ineffectiveness of dietary curcumin on the lipid profile aligns with prior research findings^50,51^.

A number of articles explore the possible impact of curcumin in lessening the susceptibility to atherosclerotic cardiovascular disease, a condition which became the foremost cause of death worldwide^52,53^. It is worth highlighting that our investigation stands out as the first such exploration towards establishing the true impact of curcumin on the susceptibility to atherosclerotic cardiovascular disease (ASCVD) within the realm of a clinical trial involving human subjects. This was done by analyzing the risk scores and classes before and after giving curcumin dosage. For the risk assessment, the method used was the pooled cohort equation as specified by the American College of Cardiology based on SBP, DBP, TC, LDL-C and HDL-C. Our findings showed a marked reduction in the ASCVD risk class following intervention with curcumin compared to the control group. Most of this decline could be attributed to the substantial decrease in SBP, DBP and LDL-C alongside the increased HDL-C levels. Consequently, by these slight changes in the crucial variables that collectively impact on the overall risk profile, the risk class of ASCVD was lowered.

Atherosclerosis is not solely linked to an increase in concentration of cholesterol, it also refers to the formation of atherosclerotic plaques, that occur as a result of various physiological mechanisms such as inflammation and oxidative stress. In this regard, TNF-alpha eventually comes out as a pivotal biomarker of inflammation that is strongly associated with atherosclerosis formation and subsequent ASCVD development. The current literature also does not fully support the prevailing hypothesis about the potential impact of curcumin in modulating inflammation associated with atherosclerosis.

Some scientific studies reveal that curcumin possesses the capability to lower the level of TNF-alpha^54,55^ while contrasting research outcomes introduce a contradictory picture by indicating a lack of statistically significant reduction in TNF-alpha concentrations subsequent to curcumin administration^56^. However, our research contributes to this discourse, concluding that curcumin has the capacity to modulate inflammation by attenuating TNF-alpha levels.

Moreover, within the domain of oxidative stress, MDA emerges as an aldehyde product of the peroxidation of polyunsaturated fatty acids of cellular membranes and reflects the levels of oxidative damage and cellular stress. Although there have been conflicting findings from numerous scientific studies and academic debates over curcumin’s effects on MDA levels, some evidence suggests that curcumin may be helpful in reducing MDA concentration^57,58^. On the contrary, alternative research outcomes offer a different perspective, indicating that the levels of MDA remain relatively unchanged and comparable both before and after curcumin administration^59^. The outcomes of our investigation suggest that, although there was no statistically significant difference between the two groups, there was a discernible reduction in MDA levels within the curcumin group when comparing pre- and post-curcumin administration, which indicates a potentially advantageous effect in mitigating oxidative stress.

Based on the documented adverse reactions associated with the administration of curcumin, there arose a need to meticulously ascertain the potential side effects of this substance, encompassing manifestations such as nausea, headache, dermatological manifestations like skin rash, gastrointestinal disturbances including diarrhea, as well as alterations in stool color to yellow^20,60^.

Following the systematic collection of the annotated compendium outlining the aforementioned adverse effects on a monthly basis from each participant enrolled in the study, an in-depth analysis revealed non-significant differences across the two groups. It was observed, however, that a small subset of patients reported incidents of nausea, headache and yellow stool. Nevertheless, it is imperative to underscore that in light of the lack of statistical significance, these observed side effects can be deemed as relatively manageable and tolerable. Consequently, curcumin was deemed safe and well-tolerated throughout the duration of the study.

Lastly, even though our study lasted 14 weeks, which is longer than the typical duration of other clinical trials involving curcumin, which generally range from 8 to 12 weeks, we advocate for future research to extend to a longer duration. Furthermore, this extension may potentially elicit more pronounced beneficial effects or long-term consequences of curcumin that are yet to manifest in relation to curcumin’s influence on variables that have not demonstrated statistically significant differences.

## Conclusion

The analysis of clinical parameters has revealed that following a duration of 14 weeks during which individuals were subjected to a daily dosage of 1500 mg of curcumin supplementation, notable changes were observed in various health markers. These changes included a decrease in SBP, DBP, LDL-C, TNF alpha levels, MDA levels, and ASCVD risk class. Conversely, an increase was noted in HDL-C levels. However, no significant modifications were observed in other assessed parameters such as BMI, HR, HbA1c, FBG, TC and TG.

Furthermore, the study did not reveal any statistically significant findings pertaining to the occurrence of side effects associated with curcumin intake, thereby indicating that its usage can be deemed as tolerable.

## Limitation

Despite the promising findings of this study, several limitations must be acknowledged. First, the study was conducted with a relatively small sample size, which may limit the generalizability of the results. Second, while patients were advised to maintain their usual lifestyle throughout the study, we did not formally assess for confounding lifestyle factors such as physical activity levels or dietary habits (including healthy or unhealthy food intake), both of which can significantly influence lipid profile and cardiovascular risk. Lastly, the follow-up period was relatively short, and long-term effects of curcumin administration remain unknown. Future studies with larger sample size, longer duration and better control of lifestyle variables are warranted to confirm these results.

## Data Availability

The final dataset of the trial will be available on request from the corresponding author, after obtaining the permission of the Regional Ethics Committee.
